# Non-invasive functional evaluation of lower urinary tract dysfunction in children with vesicoureteral reflux: a retrospective cohort study

**DOI:** 10.25122/jml-2026-0087

**Published:** 2026-09

**Authors:** Anna Edva Frunda, Andras Kiss, Lorand Tibor Reman, Raul Dumitru Gherasim, Réka Borka-Balas, Szabolcs Andre, Daniel Porav-Hodade, Hajnal Finta, Călin Bogdan Chibelean, Orsolya Katalin Ilona Mártha

**Affiliations:** 1 Doctoral School, George Emil Palade University of Medicine, Pharmacy, Science, and Technology of Târgu Mureș, Târgu Mureș, Romania; 2 Department of Pediatrics, Heim Pál National Institute of Pediatrics, Semmelweis University, Budapest, Hungary; 3 Department of Urology, George Emil Palade University of Medicine, Pharmacy, Science, and Technology of Târgu Mureș, Târgu Mureș, Romania; 4 Department of Pediatrics, George Emil Palade University of Medicine, Pharmacy, Science, and Technology of Târgu Mureș, Târgu Mureș, Romania; 5 Department of Public and Health Management,, George Emil Palade University of Medicine, Pharmacy, Science, and Technology of Târgu Mureș, Târgu Mureș, Romania

**Keywords:** vesico-ureteral reflux, lower urinary tract symptoms, urodynamics, urination disorders, ultrasonography, symptom assessment, child, BBD, bladder bowel dysfunction, EAU, European Association of Urology, ESPU, European Society for Pediatric Urology, IQR, interquartile range, LUTD, lower urinary tract dysfunction, LUTS, lower urinary tract symptoms, PLUTSS, Pediatric Lower Urinary Tract Symptom Score, PVR, post-void residual urine, Qmax, maximum urinary flow rate, SD, standard deviation, UTI, urinary tract infection, VCUG, voiding cystourethrography, VUR, vesicoureteral reflux

## Abstract

Pediatric lower urinary tract dysfunction (LUTD) is frequently underdiagnosed and may place selected patients at risk of upper urinary tract deterioration; vesicoureteral reflux (VUR) is often associated with it. This retrospective, observational, single-center study characterized non-invasive functional findings in children with primary VUR and lower urinary tract symptoms and assessed how uroflowmetry, post-void residual urine (PVR), ultrasound, and the Pediatric Lower Urinary Tract Symptom Score (PLUTSS) contribute to decision-making before invasive treatment. Forty-six children aged 5 to 17 years were evaluated between January 2021 and December 2025 at Marmed Ambulatory, Târgu Mureș, Romania. VUR was diagnosed by voiding cystourethrography and graded I to IV according to the International Reflux Grading System. Thirty children (65.22%) were girls; mean age was 10.68 ± 5.11 years (median 11.0; IQR 6.8–16.0). Storage symptoms predominated (*n* = 23, 50.00%) over voiding symptoms (*n* = 18, 39.13%), with combined symptoms in 5 children (10.87%). Kidney ultrasound was abnormal in 17 children (36.96%) (hydronephrosis, cyst, duplication, or solitary kidney) and bladder ultrasound in 8 (17.39%) (diverticula or wall thickening >5 mm). Bladder capacity was reduced in 15 children (32.61%), normal in 20 (43.48%), and increased in 11 (23.91%). Uroflowmetry curves were continuous bell-shaped in 27 children (58.70%), continuous plateau in ten (21.74%), and interrupted in nine (19.57%); mean PVR was 28 ± 25.5 mL. Non-invasive functional assessment is an important first-line diagnostic approach in children with VUR and LUTD, and identifying and treating bladder dysfunction before invasive reflux correction may improve patient selection and treatment outcomes.

## INTRODUCTION

Lower urinary tract symptoms (LUTS) are frequently encountered in children, and daytime lower urinary tract dysfunction (LUTD) has a reported prevalence of 1–20% [1]. LUTD comprises a spectrum of storage (filling phase) and voiding (emptying) dysfunctions, including urgency, frequency, incontinence, enuresis, and dysfunctional voiding [1]. Persistent dysfunction may predispose to recurrent urinary tract infections (UTIs), vesicoureteral reflux, and renal scarring in susceptible patients.

Vesicoureteral reflux (VUR), the retrograde flow of urine from the bladder to the upper urinary tract, usually during voiding, may be unilateral or bilateral and may be asymptomatic. It is frequently associated with nephropathy and with bladder and bowel dysfunction, including detrusor overactivity, urinary urgency, urge incontinence, hypoactive bladder, postponement of urination, and other voiding disturbances [2,3]. Several publications have reported the coexistence of VUR and LUTD in children, although whether this dysfunction persists long term remains uncertain [4–6].

Four options are currently available for managing primary VUR in infants and children: surveillance or intermittent treatment of UTIs with management of bladder and bowel dysfunction; continuous antibiotic prophylaxis; endoscopic subureteral injection of tissue-augmenting substances; and ureteral reimplantation by open, laparoscopic, or robot-assisted surgery [1]. Current debate centers on when to perform diagnostic imaging and how best to identify the children who will benefit from continuous antibiotic prophylaxis or surgical intervention [1]. Urodynamic and video-urodynamic studies may be considered in older, toilet-trained children with VUR who have persistent urinary dysfunction or who fail prophylactic therapy [4,7,8]. Children with VUR and coexisting bladder or bowel dysfunction are at higher risk of infection despite continuous prophylaxis, show a substantially lower rate of spontaneous resolution (31% versus 61%), and have reduced success rates after endoscopic correction [7]. Such children may show uninhibited detrusor contractions or detrusor sphincter dyssynergia, which should be identified and treated before surgical intervention is considered [7].

Treatment of bladder and bowel dysfunction, including timed voiding, laxatives, pelvic floor therapy and exercises, behavioral modification, and anticholinergic therapy, reduces voiding symptoms, improves bladder function, and increases the rate of spontaneous resolution [5,9–11]. Where VUR is associated with LUTD, the diagnostic workup must therefore address both conditions.

This study aimed to characterize non-invasive functional findings in children with primary VUR and associated LUTD and to evaluate how uroflowmetry, post-void residual urine, ultrasound, and PLUTSS contribute to clinical decision-making before invasive treatment in a single-center cohort evaluated between January 2021 and December 2025.

## MATERIAL AND METHODS

### Study design and setting

This retrospective observational cohort study included 46 children with primary grade I–IV VUR evaluated for LUTD at a single center, the Urodynamic Unit of Marmed Ambulatory, Târgu Mureș, Romania, between January 2021 and December 2025. VUR was diagnosed by voiding cystourethrography and graded according to the International Reflux Grading System. The study was performed after informed consent was obtained from the children’s parents.

### Participants

Inclusion criteria were confirmed primary VUR (grades I–IV), age 5–17 years, the presence of at least one lower urinary tract symptom, and completion of the Pediatric Lower Urinary Tract Symptom Score (PLUTSS) questionnaire. Children with neurogenic bladder, posterior urethral valves, a history of lower urinary tract reconstructive surgery, an active urinary tract infection at the time of evaluation, or incomplete medical records were excluded.

### Clinical assessment and definitions

A complete history was taken and a physical examination performed, followed by abdominal ultrasound and uroflowmetry. LUTS were defined as the presence of at least one storage or voiding symptom: decreased (≤4 times/day) or increased (≥7 times/day) voiding frequency, urgency, urinary incontinence, dysuria, hesitancy, straining, a poor or interrupted urine stream, terminal dribbling, incomplete bladder emptying, holding maneuvers, or nocturnal enuresis. Symptoms were quantified using PLUTSS, comprising 13 symptom items and one quality-of-life item; only children with a completed questionnaire were included. Age at the start of toilet training, previous episodes of UTI, and family history of enuresis were also recorded. In selected cases, a three-day voiding diary was completed to record fluid intake, urine output, and the number and volume of wetting episodes. Urinalysis and urine culture were obtained for every child before entry.

### Imaging and uroflowmetry

Kidney and bladder ultrasonography was performed in all children using 7.5-MHz linear and 5-MHz convex probes (Consona N8, Mindray, Shenzhen, China), assessing bladder configuration, bladder wall thickness, and post-void residual (PVR) urine volume. Uroflowmetry was performed using the Solar Blue system (MMS-Laborie, Enschede, the Netherlands) under physiological conditions, standing for boys and sitting for girls, with a voided volume of at least 150 mL wherever possible; in younger children and those with reduced functional bladder capacity, lower voided volumes were accepted but interpreted with caution. Interpretation covered curve pattern, maximum urinary flow rate (Qmax), duration of micturition, hesitancy, time to Qmax, and voided volume. Invasive urodynamic studies were performed only where clinically indicated, in two children with suspected detrusor sphincter dyssynergia.

### Statistical analysis

Continuous variables are summarized as mean ± SD and median [IQR], and categorical variables as *n* (%).

## RESULTS

Of the 46 children with VUR, 18 had grade I, 15 grade II, 11 grade III, and 2 grade IV. Storage symptoms predominated (*n* = 23, 50.00%) over voiding symptoms (*n* = 18, 39.13%), with combined symptomatology, mostly storage, in five children (10.87%; Table 1). Abnormal kidney ultrasound findings were present in 17 children (36.96%): hydronephrosis in 13, all with grade III–IV VUR; a 3 cm solitary cyst in one girl; duplication in two; and a solitary kidney in one child with grade I VUR. Abnormal bladder ultrasound findings were present in eight children (17.39%): diverticula in two and bladder wall thickening >5 mm in six (13.04%). Bladder capacity was reduced in 15 children (32.61%), normal in 20 (43.48%), and increased in 11 (23.91%); of the latter, eight — all girls — had a capacity above 300 mL without residual urine (Table 2). Mean post-void residual urine volume was 28 ± 25.5 mL. Uroflowmetry curves were continuous bell-shaped in 27 children (58.70%), continuous plateau in 10 (21.74%), and interrupted in 9 (19.57%). Qmax was ≥15 mL/s in 12 children (26.09%) and below 10 mL/s in nine (19.57%) (Table 2). Terminal dribbling was present in five children (10.87%); it was recorded as a voiding symptom and not as a uroflowmetry curve pattern. Symptom categories were therefore not mutually exclusive, and the cumulative number of findings may exceed the study population (*n* = 46).

**Table 1:** Demographic characteristics and lower urinary tract symptoms of the study population (*n* = 46)

Variable	*n* (%)
**Age (years)**	
Mean ± SD	10.68 ± 5.11
Median [IQR]	11.0 [6.8–16.0]
5–7	21 (45.65)
8–11	10 (21.74)
12–17	15 (32.61)
**Sex**	
Female	30 (65.22)
Male	16 (34.78)
**Storage symptoms**	
Non-monosymptomatic enuresis	11 (23.91)
Urgency, wetting on the way to the toilet	25 (54.35)
Increased voiding frequency (≥7 times/day)	33 (71.74)
Decreased voiding frequency (≤4 times/day)	13 (28.26)
**Voiding symptoms**	
Interrupted voiding	11 (23.91)
Sensation of incomplete emptying	16 (34.78)
Straining during voiding	6 (13.04)
Pain during voiding	6 (13.04)
**Bowel symptoms**	
Constipation	9 (19.57)

IQR, interquartile range; SD, standard deviation. Children could present with more than one symptom, so symptom categories are not mutually exclusive, and the cumulative number of symptoms exceeds the number of patients. Percentages refer to the proportion of the 46 patients presenting each individual finding.

**Table 2:** Clinical and non-invasive functional findings by PLUTSS score (*n* = 46)

Variable	*n* (%)	PLUTSS score
**Bladder capacity**		
Normal	20 (43.48)	15.08 ± 1.4
Reduced	15 (32.61)	25.06 ± 1.6
Increased	11 (23.91)	17.38 ± 1.45
Post-void residual, mean (mL)	28 ± 25.5	15.45 ± 1.7
**Qmax (mL/s)**		
≤10	9 (19.57)	16.70 ± 2.2
10.1–14.9	25 (54.35)	17.61 ± 1.8
≥15	12 (26.09)	19.46 ± 2.1

PLUTSS, Pediatric Lower Urinary Tract Symptom Score; Qmax, maximum urinary flow rate.

PLUTSS scores varied with bladder capacity. Children with reduced bladder capacity had significantly higher PLUTSS scores than those with normal bladder capacity (25.06 ± 1.6 vs. 15.08 ± 1.4), corresponding to an approximately 10-point difference. Children with increased bladder capacity had an intermediate mean PLUTSS score of 17.38 ± 1.45.

Associated conditions were constipation in nine children (19.57%), hypospadias in three, attention deficit hyperactivity disorder in two, phimosis in two boys, and meningocele in two.

Treatment was tailored to the functional findings. Children with irregular curve patterns received VUR treatment combined with behavioral therapy, bladder training, or pharmacotherapy (alpha-1 blockers, anticholinergics), with invasive treatment where bladder outlet obstruction was confirmed. Children with normal functional findings received VUR treatment alone: continuous antibiotic prophylaxis, endoscopic subureteral injection of tissue-augmenting substances, or ureteral reimplantation.

## DISCUSSION

Lower urinary tract dysfunction and vesicoureteral reflux are closely interconnected conditions in pediatric urology. Numerous studies have shown that bladder dysfunction contributes not only to recurrent urinary tract infections but also to the persistence of reflux and to reduced success rates of conservative and surgical treatment [2,3,5,7]. Every child in the present cohort had VUR together with lower urinary tract symptoms — a consequence of the inclusion criteria rather than an observed association — which underlines the need for structured functional evaluation before therapeutic decisions are taken [4,10].

The most frequent clinical manifestations were urgency, increased daytime frequency, and non-monosymptomatic enuresis, and storage symptoms predominated over voiding symptoms. Similar observations have been reported previously, with detrusor overactivity identified as the predominant functional abnormality in children with VUR. Abnormal bladder filling may therefore contribute to sustained elevated intravesical pressure, although bladder pressure was not measured in this study.

Non-invasive functional assessment proved informative in our cohort. Uroflowmetry, combined with post-void residual urine volume and ultrasound, identified abnormalities that were not apparent from the clinical history. Bell-shaped curves were the most common pattern; plateau and interrupted curves were also observed, suggesting dysfunctional voiding or bladder outlet obstruction in a subset of children. These findings support uroflowmetry as an integral component of the routine evaluation of toilet-trained children with VUR and LUTD rather than an optional additional examination.

Ultrasound also contributed useful information, with abnormal upper urinary tract findings in 17 children (36.96%) and abnormal bladder findings in 8 (17.39%). Although ultrasonography cannot replace functional studies, it characterizes anatomy and, together with post-void residual measurement, allows non-invasive assessment of bladder emptying.

Constipation was identified in nine children (19.57%). The interaction between bladder and bowel function is well described, and constipation is recognized as a risk factor for urinary urgency, dysfunctional voiding, and recurrent urinary tract infections, and as a cause of delayed resolution of VUR [6,7]. Bowel management should therefore form an essential component of LUTD treatment in children with reflux.

Our results are consistent with current European Association of Urology and European Society for Pediatric Urology (EAU/ESPU) guidelines, which recommend systematic identification and treatment of bladder and bowel dysfunction in children with LUTD, including urotherapy, timed voiding, treatment of constipation, pelvic floor rehabilitation, and pharmacological therapy where indicated [1]. Bladder dysfunction should be recognized and managed before invasive correction of reflux wherever possible; this does not imply that invasive treatment requires normal functional findings.

This study has several limitations. Its retrospective, single-center design and relatively small sample of 46 children may limit the generalizability of the findings. The analysis was primarily descriptive, and no multivariable analyses were performed. Treatment outcomes and long-term follow-up were not evaluated, preventing any assessment of the prognostic value of the functional abnormalities identified. The completeness of several functional parameters, including Qmax and PVR, varied between patients, and some clinical data were drawn from free-text medical records. Treatment information was recorded using multiple labels without standardized time points, precluding robust outcome analysis. Future prospective studies should incorporate standardized symptom scores, validated definitions, and longitudinal follow-up.

## CONCLUSION

Non-invasive functional assessment, including uroflowmetry, ultrasound, post-void residual urine, and PLUTSS, represents an important first-line diagnostic approach in children with VUR and LUTD. Identifying and treating bladder dysfunction before invasive reflux correction may improve patient selection and treatment outcomes.

## Data Availability

The de-identified dataset supporting the findings of this study, including demographic, clinical symptom, ultrasound, uroflowmetry, and PLUTSS data, is available from the corresponding author upon reasonable request, subject to approval by the Ethics Committee of Policlinica Marmed.

## Personal thanks

This study forms part of the doctoral thesis of E.A. Frunda at the Doctoral School of the George Emil Palade University of Medicine, Pharmacy, Science, and Technology of Târgu Mureș, Romania.
